# Lewis Pair Catalysts in the Polymerization of Lactide and Related Cyclic Esters

**DOI:** 10.3390/molecules23010189

**Published:** 2018-01-17

**Authors:** Xinlei Li, Changjuan Chen, Jincai Wu

**Affiliations:** 1 State Key Laboratory of Applied Organic Chemistry, College of Chemistry and Chemical Engineering, Key Laboratory of Nonferrous Metal Chemistry and Resources Utilization of Gansu Province, Lanzhou University, Lanzhou 730000, China; lixl16@lzu.edu.cn (X.L.); chenzhj15@lzu.edu.cn (C.C.); 2 College of Chemistry and Pharmaceutical Engineering, Huanghuai University, Zhumadian 463000, China

**Keywords:** Lewis pair, polymerization, lactide and lactones

## Abstract

Polyesters, especially poly(lactide) (PLA), are used widely as biodegradable and biocompatible materials, yet their controllable synthesis, especially the stereoselective synthesis of polyesters, is still a challenge. Recently some excellent Lewis pair catalysts for ring-opening polymerization (ROP) of lactide and related cyclic esters have emerged. This review article will highlight the key advances in the ROP catalyzed by Lewis pair compounds with the aim of encouraging the wider application of Lewis pair catalysts in the polymerization of lactide and related cyclic esters.

## 1. Introduction

Over the past three decades, biodegradable and biocompatible polyesters and their copolymers have attracted considerable attention due to their wide applications in packaging, agricultural, and new biomedical and pharmaceutical fields. The starting materials for these polyesters, for example poly(lactide) (PLA), are derived from annually renewable resources such as corn, beet, and others. Due to the concerns about our environment, global pollution, and the depletion of petrochemical feedstocks, renewable and environmentally friendly polyesters are becoming increasingly important for a sustainable future. A particularly convenient method for the synthesis of polyesters is the ring-opening polymerization (ROP) of cyclic esters due to its advantages of producing polymers with well controlled molecular weight and narrow molecular weight distribution (*Đ*). Various types of catalysts have been developed for the synthesis of polyesters, especially for polylactide. Metal complexes usually exhibit high activities and outstanding (stereo)selectivities [1,2,3], but the applicability of these complexes is frequently not broad, and tailor-made solutions have to be designed for different monomers; in addition, most of these metal complexes are sensitive to air/water which limits their industrial applications. Organocatalysts can display higher tolerances under different catalytic conditions [4]. However, there is still no single organocatalyst able to polymerize the full range of lactides and lactones, and the activity of organocatalysts is generally much lower than that of the different metal complexes. Many chemists have focused on the development of new catalysts for exquisitely controlling chain growth, stereochemistry, and even the topological structure of polyesters. As a new strategy of combining the respective advantages of both metal complex catalysts and organocatalysts, Lewis pair complexes have emerged recently as new kind of catalyst, and we focus on this topic in this review. Bifunctional organocatalysts including Brønsted acids and bases, which has been reviewed recently [5,6] were not included here. In these bifunctional organocatalyst systems of Brønsted acid and base, the carbonyl of a lactide monomer can be activated via hydrogen bonding with a Brønsted acid and the initiating/propagating alcohol can be activated via the Brønsted base.

**Scheme 1:**
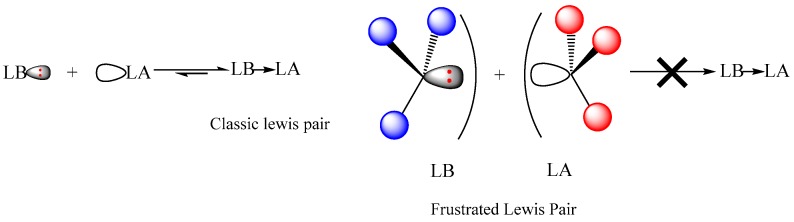
Active classic Lewis pair and frustrated Lewis pair catalysts.

Usually, active Lewis pair catalysts need a separated Lewis base (LB) and Lewis acid (LA) in a frustrated Lewis pair due to a steric hindrance or via a dissociative equilibrium between Lewis acid and base in a classic Lewis pair (Scheme 1). Due to the combination of unquenched acid and base activities, the Lewis pair can activate cyclic esters using the Lewis acid and the Lewis base can activate initiating/propagating alcohol via the hydrogen bonding to make it nucleophilic enough to attack the monomer or directly play as an initiator to attack the monomer; then the polymerization of cyclic esters mediated by these Lewis pair complexes can proceed smoothly.

## 2. Zinc Complex/Base Lewis Pairs Catalysts

**Scheme 2:**
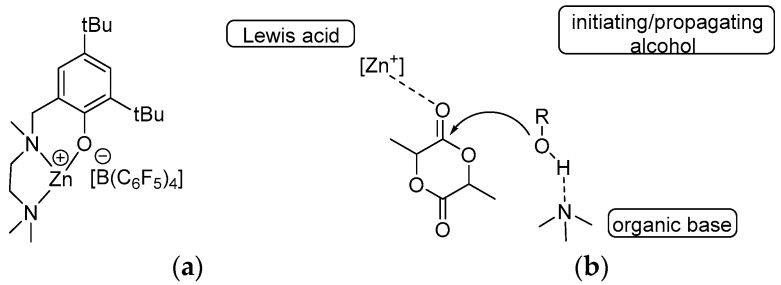
(**a**) Cationic zinc complex [{NNO}Zn]^+^[B(C_6_F_5_)_4_)]^−^; (**b**) Bifunctional activation of the monomer (by the Lewis acidic zinc complex (**1**)) and initiating/propagating alcohol (by the amine) proposed to account for the dual ROP of lactide.

In 2011, Guillaume, Bourissou, and co-workers reported that Lewis pairs of a discrete cationic complex [{NNO}Zn]^+^[B(C_6_F_5_)_4_)]^−^ (Lewis acid) and pentamethylpiperidine (PMP, as Lewis base) can efficiently promote the ROP of lactide under mild conditions (Scheme 2) [7]. Using *neo*-PentOH (*neo*-pentanol) as an initiator, complete conversion of the monomer in up to 98% yield can be achieved within 3 h at room temperature in CH_2_Cl_2_ with desirable polylactide M_n_ values of up to 14,500 g/mol and molecular weight distributions of *Đ* values ranging between 1.2 and 1.4. Whereas, the polymerization cannot happen in the presence of *neo*-PentOH with only [{NNO}Zn]^+^[B(C_6_F_5_)_4_)]^−^ or only PMP, revealing the cooperativity of Lewis acid and Lewis base. In addition, a broad range of reactivities was observed depending on the basicity of the amines: a rapid polymerization is observed with PMP (p*K*_a_ = 11.2), but hardly any polymerization can occur with PhNMe_2_ (p*K*_a_ = 5.1) as Lewis base, which suggests the initiating/propagating alcohol is activated by the amine Lewis base. As a result, it was proposed that the electrophilic zinc cation activates the monomer by coordination to the carbonyl moiety while the amine activates the initiating/propagating alcohol through hydrogen bonding (Scheme 2). No epimerization of the monomer and polymer chain occur, however the PLA samples derived from *rac*-lactide were found to be essentially atactic, indicating the absence of significant stereo chain-end control.

**Scheme 3:**
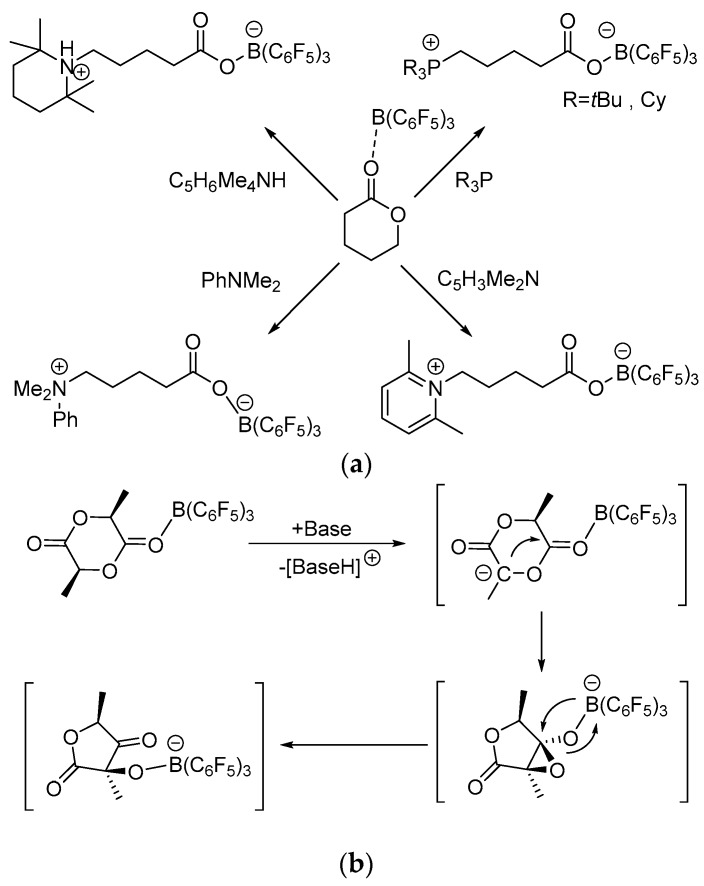
(**a**) ring-opening of δ-valerolactone; (**b**) Proposed mechanism of lactide ring contraction.

**Scheme 4:**
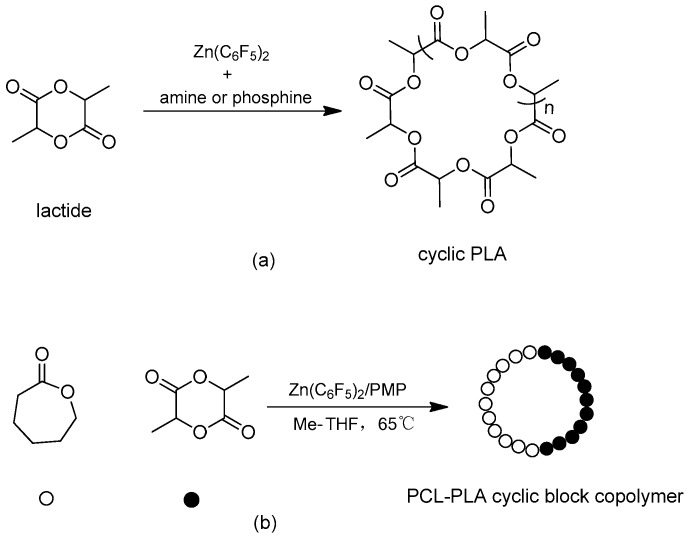
(**a**) ROP of lactide with Zn(C_6_F_5_)_2_-based Lewis pairs; (**b**) Sequential polymerization of *ε*-CL and lactide affording PCL-PLA cyclic block copolymer.

Lewis pairs of the combination of B(C_6_F_5_)_3_ and phosphine or N-bases can mediate the ring-opening of δ-valerolactone (δ-VL) affording zwitterionic species as reported by Stephan and co-workers (Scheme 3a) [8]. Because the treatment of B(C_6_F_5_)_3_ and δ-VL can form an adduct of δ-VL-B(C_6_F_5_)_3_, the ring-opening reaction is thought to result from the Lewis acid activation of carbonyl bond of δ-VL, which prompts the attack by the Lewis base. However, only stoichiometric ring-opening reactions were reported. The reaction of lactide only gives the ring contraction product (Scheme 3b). It is interesting that when B(C_6_F_5_)_3_ is replaced with Zn(C_6_F_5_)_2_, the combination of Zn(C_6_F_5_)_2_ and organic bases (amines or phosphines) can promote the controlled ring-opening polymerization of lactide (Scheme 4a) and ε-caprolactone (ε-CL), affording polymers of cyclic architecture, as reported by Amgoune, Bourissou, and co-workers [9]. Efficient chain-extension can give access to cyclic block copolymers of PLA−PCL (Scheme 4b). Such systems offer a new route to cyclic polyesters. The cyclic topology of the polymer was assigned by a combination of Mark-Houwink plots and MALDI-TOF, the latter of which showed no trace of linear products. ^1^H-NMR analysis also revealed perfectly isotactic PLA with no end group signals, indicating that this Lewis pair does not induce epimerization of l-lactide.

**Scheme 5:**
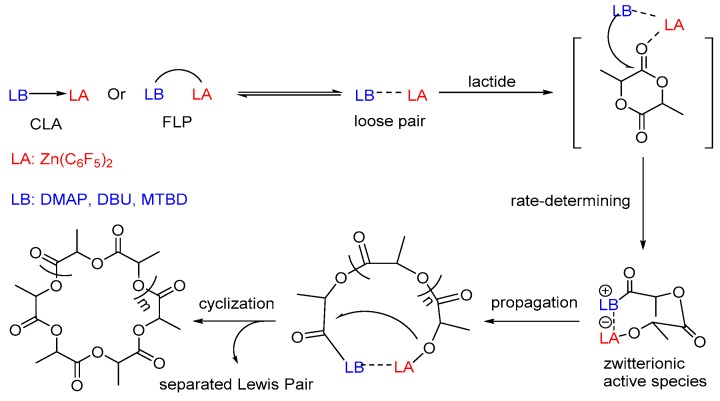
Proposed mechanism for the ring-opening polymerization of lactide catalyzed by Lewis pairs.

The mechanism of the above system was further explored in details by Li and co-workers in 2016 [10]. Combination of Zn(C_6_F_5_)_2_ with DMAP (4-dimethylaminopyridine), NHC (1,3-bis(2,4,6-trimethylphenyl) imidazole-2-yli-dene-(MesNHC)), DBU (1,8-diazabicyclo[5,4,0]undec-7-ene) or MTBD (7-methyl-1,5,7-triazabi-cyclo[4,4,0]decane-5-ene) can afford Lewis pairs with different interaction modes. The reactivity of the Lewis adduct Zn(C_6_F_5_)_2_/DMAP was quenched significantly because the combination of DMAP with Zn(C_6_F_5_)_2_ exclusively formed a classical Lewis adduct (CLA); but the Lewis acidity of Zn(C_6_F_5_)_2_ was not quenched completely, and the polymerization can be initiated at a high temperature. When bulky MTBD and DBU were used, the Lewis pairs Zn(C_6_F_5_)_2_/DBU and Zn(C_6_F_5_)_2_/MTBD exhibited relatively high activity at room temperature because of the weaker interaction between Zn(C_6_F_5_)_2_ and MTBD or DBU for the larger steric hindrance. The investigation into the polymerization behavior of lactide showed that the weak interacting Lewis pair of Zn(C_6_F_5_)_2_/MTBD exhibited much higher activity in the polymerization of lactide and much lower temperature dependence compared with the Lewis adduct Zn(C_6_F_5_)_2_/DMAP. And a possible bifunctional activation mechanism was proposed as shown in Scheme 5. The Lewis acidity of Zn(C_6_F_5_)_2_ was neutralized completely in CLA, whereas it was quenched partly in weak interacting Lewis pairs of Zn(C_6_F_5_)_2_/DBU and Zn(C_6_F_5_)_2_/MTBD. With increasing temperature, the interaction between the Lewis acid and the Lewis base in CLA become seriously impaired, thus the Lewis acidity of Zn(C_6_F_5_)_2_ was recovered. Lactide would be activated electronically by coordination to the Lewis acid Zn(C_6_F_5_)_2_. Simultaneously, an amine attacked nucleophilically the carbonyl of lactide, forming a zwitterionic active species with Zn(C_6_F_5_)_2_ in one end and an amine in the other end. The Zn(C_6_F_5_)_2_ moiety associated with the amine moiety in the active species, and they work cooperatively to activate the lactide monomer in the propagation process. The polymerization was terminated by the nucleophilic attack of the terminal alkyloxide on the carbonyl, yielding a cyclic polymer and releasing separated Lewis pairs.

**Scheme 6:**
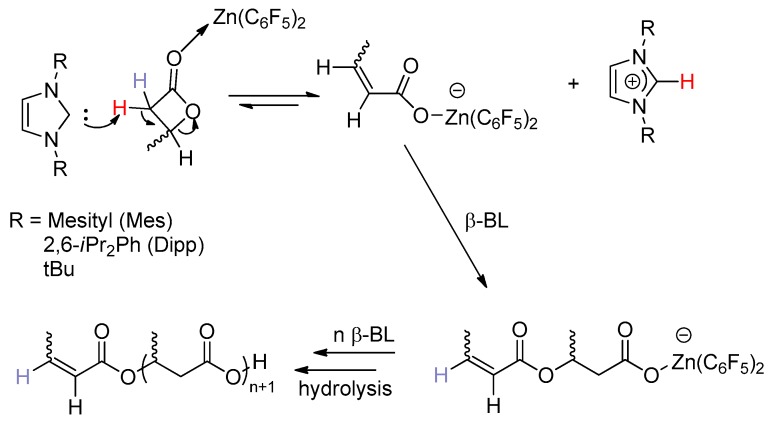
Proposed anionic mechanism for the ROP of β-BL initiated by adducts of NHC-Zn(C_6_F_5_)_2_.

Adducts of *N*-heterocyclic carbenes (NHCs) and Zn(C_6_F_5_)_2_, reported by Dagorne and co-workers [11], can mediate the ring-opening polymerization of β-BL (β-Butyrolactone) at 90 °C in toluene to afford polybutyrolactone (PBL) with narrow molecular distributions (1.17–1.22) and slightly lower molecular weights. MALDI-TOF mass spectrometric and ^1^H-NMR spectroscopic data proved the polymer chain ends are crotonates, indicating that NHC acts as a base to deprotonate β-BL to form crotonate zinc intermediate by alkyl cleavage and then initiate the ROP of β-BL via an anionic mechanism (Scheme 6). In this system, the role of Zn(C_6_F_5_)_2_ cannot be neglected, because PBLs obtained with the related free NHCs as catalysts exhibit broader *Đ* values (1.33–1.56) than those produced with adducts of NHC/Zn(C_6_F_5_)_2_ under identical conditions. The same NHC–Zn(C_6_F_5_)_2_ adducts were also found to ring-open polymerize *rac*-LA at room temperature; however, they afforded broadly dispersed and poor chain length-controlled PLA, indicative of ill-defined ROP processes. Very interestingly, the simple binary system BnOH and Zn(C_6_F_5_)_2_ generated in situ upon mixing BnOH with Zn(C_6_F_5_)_2_ was observed to polymerize *rac*-LA in a controlled and immortal manner to produce chain-length-controlled PLA. In this system Zn(C_6_F_5_)_2_ acts as Lewis acid and BnOH acts as a nucleophilic chain-transfer agent, and the ROP is proposed to via a monomer activated mechanism in which the activation of monomer is very important. Thus, it seems that the lewis acid Zn(C_6_F_5_)_2_ is more important for both systems. However, the BnOH/Zn(C_6_F_5_)_2_ initiating system displayed no activity in the ROP of β-BL (90 °C, toluene, 20 h).

## 3. Aluminum Complexes/Base Lewis Pairs Catalysts

**Scheme 7:**
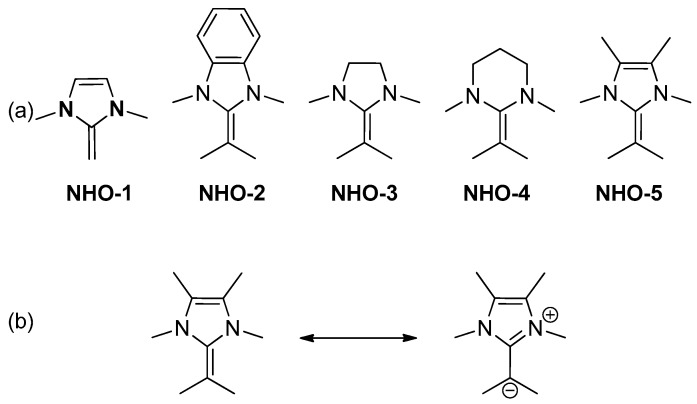
(**a**) Different NHOs; (**b**) Mesomeric formulation for NHO-**5**. Upon charge separation, an aromatic imidazolium moiety forms, supposedly explaining the high reactivity of this compound.

**Scheme 8:**
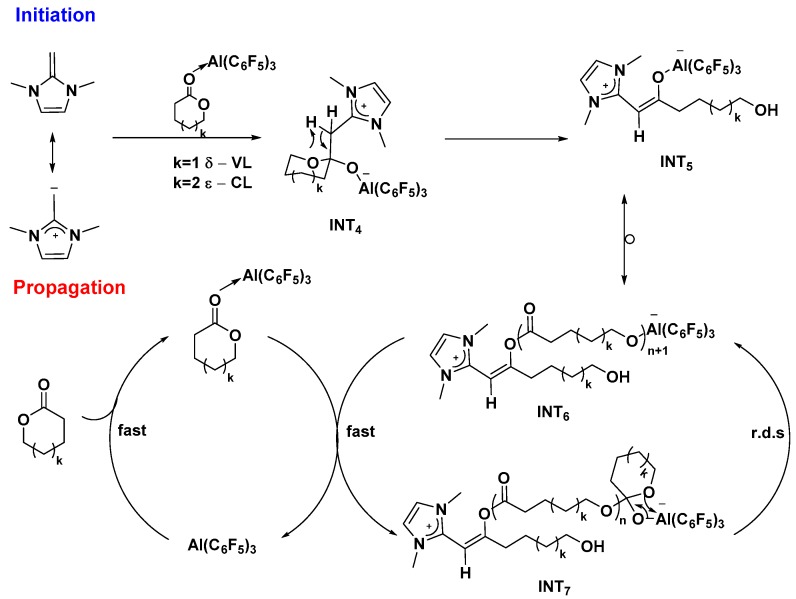
Proposed mechanism for ROP of δ-VL or ε-CL by NHO-1/Al(C_6_F_5_)_3_ Lewis pairs (Adapted with permission from ref. 24. Copyright @ 2017 American Chemical Society).

Chen et al. reported that several Al(C_6_F_5_)_3_-based Lewis pairs show high activity and effectiveness for the polymerization of conjugated polar alkenes such as methyl methacrylate (MMA), acrylamides, α-methylene-γ-butyrolactones (MBL) and γ-methyl-α-methylene-γ-butyrolactone (γ-MMBL), but only P^t^Bu_3_/Al(C_6_F_5_)_3_ Lewis pairs can polymerize ε-CL to PCL at room temperature but with a broad molecular weight distribution of *Đ* = 2.76 and a low monomer conversion of only 58% even after 20 h [12]. *N*-Heterocyclic olefins (NHOs) are readily accessible and their structure is easily tuned (Scheme 7) [13,14,15,16]. Despite their success in many applications [17,18,19,20,21,22,23], these organocatalysts have been shown to be problematic for lactone polymerization, displaying either uncontrolled behavior or no reactivity toward simple lactones such as δ-VL and ε-CL. Interestingly, the same group of Chen reported the first successful Lewis pair polymerization (LPP) system for the living ROP of δ-VL and ε-CL using the combination of *N*-heterocyclic olefin (NHO-1) and Al(C_6_F_5_)_3_ [24]. In this system, high molecular weight linear (co)polyesters (M_w_ up to 855 kg/mol) are achieved, and most of them display narrow molecular weight distributions (*Đ* as low as 1.02). Based on several key crystal structures of intermediates, the polymerization is proposed to proceed with initiation involving nucleophilic attack of the Al(C_6_F_5_)_3_-activated monomer by NHO to form the zwitterionic tetrahedral intermediates, followed by its ring-opening to generate zwitterionic enolaluminate active species (Scheme 8). In the propagation cycle, this ring-opened zwitterionic species and its homologues attack the incoming monomer activated by Al(C_6_F_5_)_3_ to generate the tetrahedral intermediate, followed by the rate-determining ring-opening step to regenerate the zwitterionic species, thus re-entering into the next chain propagation cycle. Using this Lewis pair, random copolymer of ε-CL and δ-VL can be obtained in the polymerization of a mixture of two monomers; the block copolymer also can be achieved via sequential addition of two monomers respectively.

**Scheme 9:**
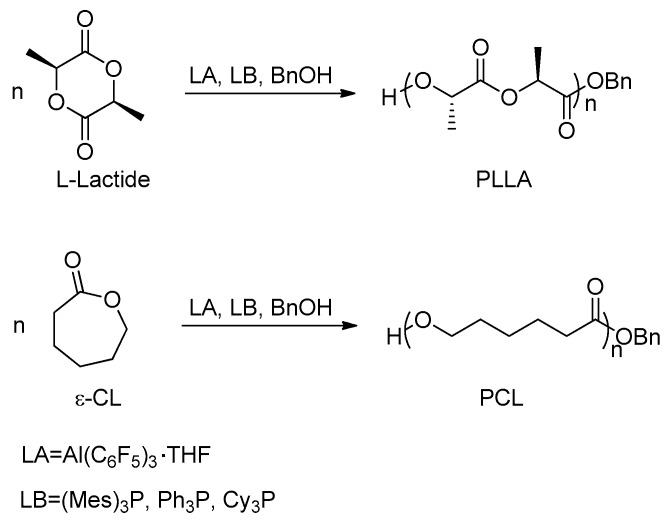
ROP of l-lactide and ε-CL catalyzed by Al(C_6_F_5_)_3_·THF-phosphine.

Al(C_6_F_5_)_3_∙THF in combination with trimesitylphosphine (Mes_3_P) or triphenylphosphine (Ph_3_P) also was utilized as catalysts to achieve a controlled polymerization of l-lactide at 100 °C by using BnOH as an initiator to produce poly(l-lactide) with narrow molecular weight distribution (*Đ* = 1.1), reported by Nakayama et al. (Scheme 9) [25]. Both the Lewis acid and the Lewis base (**LB**) were indispensable to promote the polymerization. The molecular weights of the resulting poly(l-lactide)s were controlled by the monomer to initiator feed ratio. In the Al(C_6_F_5_)_3_∙THF-Mes_3_P system, the concerted operation of Lewis acid and Lewis base in the formation of active species and/or in chain propagation also can be confirmed by the fact that no MeOH-insoluble polymer was obtained when Al(C_6_F_5_)_3_∙THF or Mes_3_P was used alone. The activities of these Al(C_6_F_5_)_3_·THF-LB Base systems also seem to be dependent on the basicity of the phosphines. When Al(C_6_F_5_)_3_∙THF-LB was replaced with B(C_6_F_5_)_3_-LB, the corresponding systems were inactive.

**Scheme 10:**
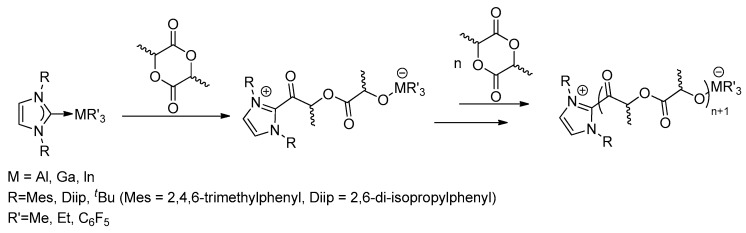
Proposed mechanism for the ROP of lactide by the group 13 metal NHC adducts of NHC-MR_3_.

Dagorne and co-workers reported that several *N*-heterocyclic carbene (NHC) group 13 metal Lewis pair adducts of NHC-MR_3_ (M = Al, Ga, In) can play as efficient initiators for the ring-opening polymerization (ROP) of *rac*-lactide (Scheme 10) [26], allowing one to obtain either linear poly(lactic acid) (PLA) or cyclic PLA. It was conjectured that the quite polar M-C carbene bond may readily dissociate in the presence of oxygen-containing polar substrates (such as cyclic esters), which are expected to be active for the ROP of lactide. The MR_3_ fragment acts as Lewis acid and the NHC moiety acts as a nucleophile to ring-open the lactide monomer. Subsequently, propagation may allow PLA chain growth from the metal center. Experimental results also showed that more sterically bulky adducts disfavor monomer access to the metal center, thus exhibit low activities. The lactide ROP behavior of the Ga(III) or In(III) adducts exhibits a moderate activity in lactide ROP but along with a poor control of the ROP process (multimodel) because of the probable involvement of several catalytically active species. The addition of an alcohol source (such as BnOH) that may act both as a nucleophile and a chain transfer agent was found to improve the control and activity of the ROP catalysis mediated by these group 13 metal adducts.

## 4. Magnesium Complex/Base Lewis Pairs Catalysts

**Scheme 11:**
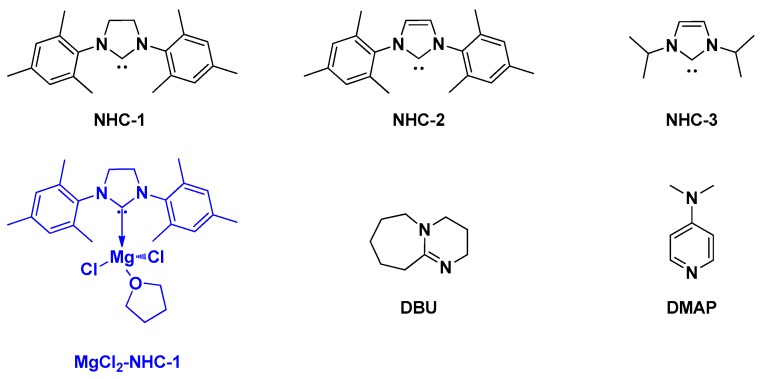
Structures of pre-catalyst MgCl_2_-NHC-**1** and all nucleophiles used in the study.

Naumann, Dove, and co-workers reported that different Lewis acids can activate different lactones to a different degree [27]. The combination of *N*-heterocyclic carbenes, 1,8-diaza-bicycloundec-7-ene (DBU) and 4-dimethylaminopyridine (DMAP) with simple Lewis acids can mediate the ROP of the macrolactone pentadecalactone (PDL) in a rapid and efficient manner. The performance of adduct of MgCl_2_-NHC-1 (Scheme 11) is comparable to a magnesium-based complex of Mg(BHT)_2_(THF)_2_ (magnesium 2,6-di-*tert*-butyl-4-methylphenoxide) for PDL polymerization [28]. Control experiments demonstrated that MgCl_2_ or free NHC-1 alone could not induce any polymerization under the same conditions (110 °C, 6 h). It was suggested that a fast and complete dissociation of NHC-1-MgCl_2_ adduct to NHC-1 and MgCl_2_ can be triggered by heating; as a result, separate addition of NHC-1 and MgCl_2_ can display a same catalytic effect for the polymerization of PDL. Remarkably, regardless of the nature of the nucleophile, the order of activity was observed to be MgX_2_ > YCl_3_ > AlCl_3_ and MgI_2_ > MgBr_2_ > MgCl_2_ in every case. The minimal influence of the organobase on polymerization activity allows for the use of simple and inexpensive precursors, for example DMAP as a Lew base is enabled to polymerize a range of monomers with a suitable co-catalyst of Lewis acid. Furthermore, extension of the study to other cyclic (di)ester monomers (of ε-CL, δ-VL, *rac*-lactide (*rac*-LA) and β-butyrolactone (β-BL) reveals the choice of Lewis acid to lead to monomer selective ROP activity and hence control over copolymer composition by choice of Lewis acid. This approach could lead to the realization of complex polymer structures with tunable physical properties from simple catalyst combinations.

## 5. NHOs/Metal Complexes Lewis Pairs Catalysts

**Scheme 12:**
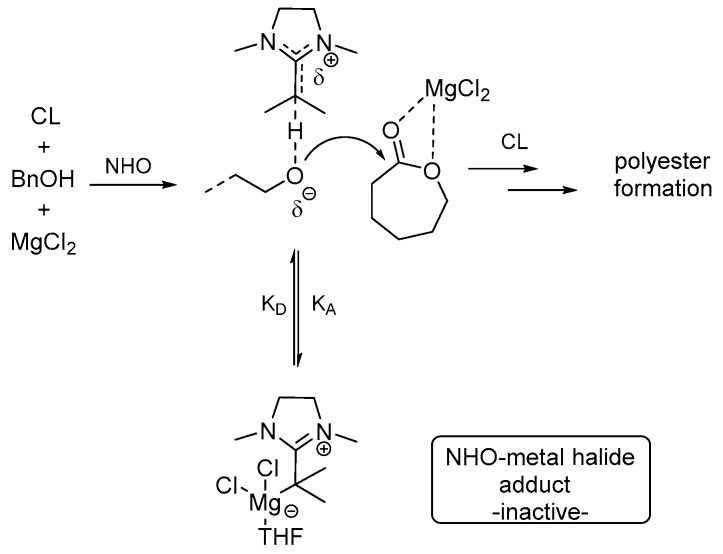
Schematic representation of the proposed polymerization mechanism, including possible adduct formation.

Naumann and co-workers utilized the cooperative interaction of four structurally different *N*-heterocyclic olefins (NHOs) (NHO-2–NHO-5, Scheme 7) with a range of simple metal halides (MgCl_2_, MgI_2_, ZnI_2_, YCl_3_, AlCl_3_, and BiCl_3_) as Lewis acidic cocatalysts for the homo- and copolymerization of ε-caprolactone (ε-CL) and δ-valerolactone (δ-VL) [29]. The single components of Lewis acids or NHOs are inactive on their own. NHO-2 can generate PCL in combination with all Lewis acids except BiCl_3_. The controlled preparation of polyesters from these monomers can be achieved, whereby desirable molecular weights and narrow molecular weight distribution (1.05 < *Đ* < 1.15) can be observed in a room temperature-based process using low catalyst loadings (0.25–0.50 mol %) for multiple combinations of NHOs and Lewis acids. The polymerization rates are strongly influenced by the nature of the involved Lewis acid. An order of activity of MgI_2_ > YCl_3_ > ZnI_2_ > MgCl_2_ is found using four Lewis bases of NHO-2 to NHO-5, which is in coordinate with the assumption that in these dual pairs the Lewis acid is the dominant part concerning the polymerization rates. In the proposed ROP mechanism (Scheme 12), the generation of Mg[I]_2_[NHO] complexes was proposed and can explain the successful application of NHO-5 for the ROP of δ-VL or ε-CL, which cannot be controlled at all by only NHO-5 as organocatalyst. Furthermore, this dual catalytic system was used to the co-polymerization of ε-CL/δ-VL. Most metal halides (such as MgI_2_, ZnI_2_, and AlCl_3_) entail δ-VL-enriched polyester, YCl_3_ favors ε-CL incorporation.

**Scheme 13:**
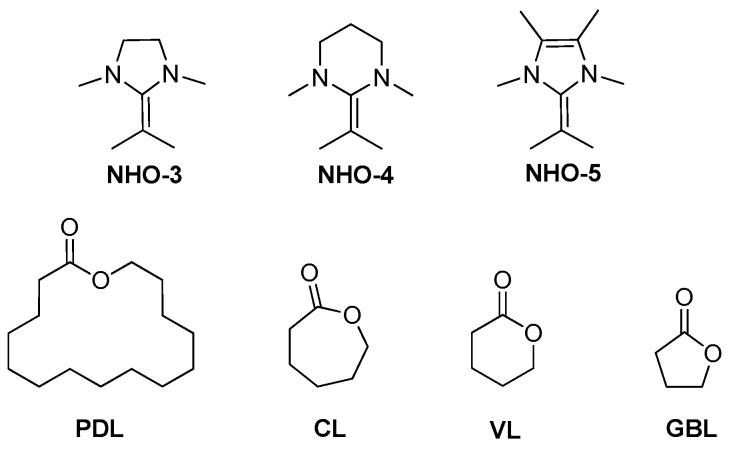
Scope of NHOs and lactone monomers.

**Scheme 14:**
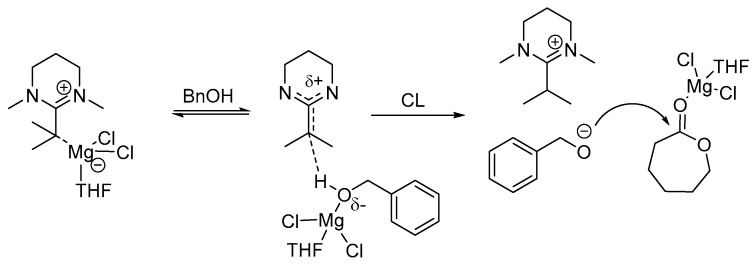
Proposed, simplified initiation mechanism for NHO Lewis pairs and lactones.

Walther and Naumann extended the application of Lewis pairs of N-heterocylic olefins (NHOs)/metal halide to less readily polymerizable latones (Scheme 13) [30]. A 16-mermered macrolactone, ω-pentadecalactone (PDL), is essentially strain-free. Consequently, PPDL synthesis commonly suffers from a broad molecular weight distribution and very limited suitable catalysts. And the copolymers of PDL with other lactones are not easily obtained too. Using some NHO/Lewis acid combinations, the polymerization of PDL can proceed smoothly. The chemical nature of the NHO seems less important than a suitable choice of the Lewis acid, which determined the success of the polymerization to a high degree. Coherently, for all cases the polymerization rates decreased in the order of MgI_2_ > MgCl_2_ > YCl_3_ > ZnI_2_. The copolymerization of PDL with ε-CL can reach high or quantitative conversion (M_n_ = 10–30 kg/mol) whereby 50% PDL content and perfectly random polymer structures were accessible. One-pot 1:1 PDL/δ-VL copolymerizations can result in high or low PDL content according different Lewis acid and reaction conditions. Notorious nonstrained γ-butyrolactone (GBL) is also less readily polymerizable latones. Although Chen and Hong co-workers succeeded in the polymerization of GBL, the polymerization conditions of low reaction temperatures (−40 °C) and high monomer concentration (10 M) are somewhat harsh [31]. Using the Lewis paired catalysts of NHOs/metal halide, the copolymerization of GBL with PDL, ε-CL, and δ-VL can be observed and the copolymerization behavior is strongly dependent on the applied Lewis pair (with slightly low GBL content (5–22%)). A simplified initiation mechanism for NHO LPs and lactones was proposed (Scheme 14): a cooperative deprotonation occurs when BnOH is mixed with NHOs and metal halides; the proton is transferred to the NHO, forming the positively charged counterion. The alcoholate will then attack the lactone monomer, which itself is activated by coordination to a Lewis acid, most probably via the carbonyl oxygen.

## 6. Boron Complex/Base Lewis Pairs Catalysts

**Scheme 15:**
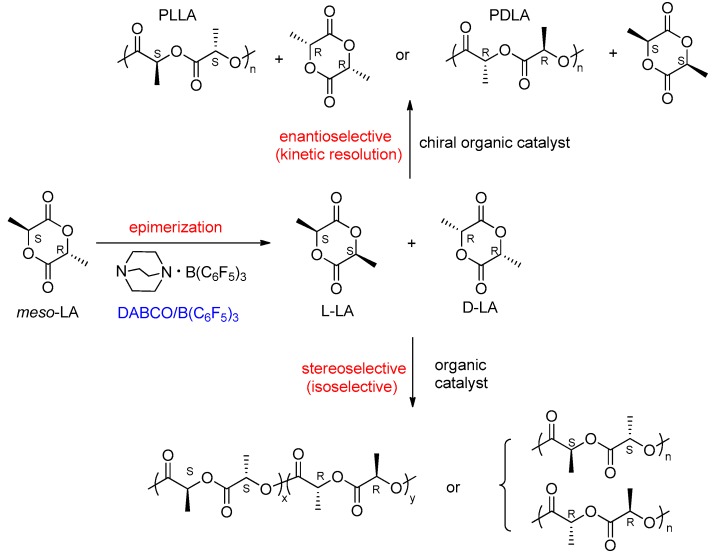
Utilization of *meso*-LA for synthesis of isotactic polylactide (Adapted with permission from ref. 32. Copyright @ 2015 American Chemical Society).

**Scheme 16:**
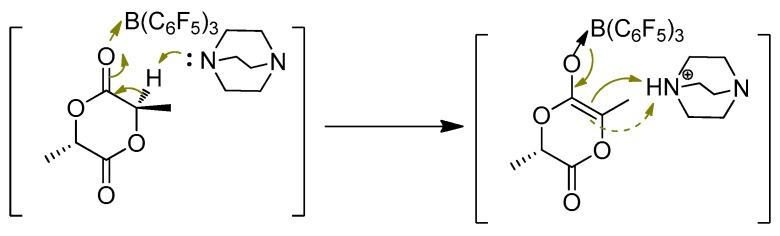
Proposed mechanism for the epimerization of *meso*-lactide.

The optical purity of l-lactide is important for current industrial production of poly(l-lactide) using the ROP method, because it can significantly affect the materials properties of final poly(l-lactide) product, such as crystallinity and biodegradation rate. While in the progresses of synthesis of l-lactide from l-lactic acid with metal complexes as catalysts or from the thermal degradation of PLLA in the feedstock recycling, some *meso*-lactide cannot be avoided as a byproduct or waste that needs to be removed. Interesting, Chen et al discovered that Lewis pairs of DABCO/B(C_6_F_5_)_3_ adduct (1,4-diazabicyclo [2.2.2]octane, DABCO) can catalyze the epimerization of *meso*-lactide (LA) quantitatively into *rac*-LA in 2015 (Scheme 15) [32]. This research seems to be a good way to solve the above serious problem of impurity of *meso*-lactide in l-LA. In this progress, it is important to shift the dynamic *meso*-LA⇔*rac*-LA equilibrium toward *rac*-LA, for example at a low temperature (room temperature); nonpolar solvent of toluene also is important, which enables the precipitation of *rac*-LA from solution of mixture once formed markedly and enhances the *meso*-to-*rac*-LA conversion up to 99%. The mechanism for this transformation of *meso*-to-*rac*-LA is presumably attributed to the cooperativity of the Lewis pairs in that B(C_6_F_5_)_3_ Lewis acid activates the substrate *meso*-LA via carbonyl coordination, which accelerates the depronation at the tertiary carbon of the activated substrate by the base of DABCO, leading the planar enolate intermediate that can be reprotonated causing epimerization (Scheme 16). Noteworthy, the performance of the preformed classical Lewis adduct (insoluble) DABCO·B(C_6_F_5_)_3_ for this epimerization reaction is identical to the separated addition of borane and DABCO, where the borane and *meso*-LA was premixed and then the addition of DABCO can start the reaction. This result indicates that this Lewis pairs adduct can reversibly and rapidly dissociate into the respective base and acid to catalyze the epimerization. The obtained *rac*-LA can further be kinetically polymerized into poly(l-lactide) and optically resolved d-LA, with a high stereoselectivity k_L_/k_D_ of 53 and an ee values of 91% at 50.6% monomer conversion, by bifunctional enantiopure chiral catalyst that incorporates three key elements (β-isocupreidine core, thiourea functionality, and chiral BINAM) into a single organic molecule.

It is intriguing that the epimerization reaction and enantioselective polymerization can be coupled into a one-pot process for transforming *meso*-LA directly into poly(l-lactide) and d-LA: authors first performed the quantitative epimerization of *meso*-LA by DABCO/B(C_6_F_5_)_3_ (0.01 mol %) in toluene, removed the solvent to give *rac*-LA/*meso*-LA in a ratio of 99/1, and then added enantiopure organic catalyst in *o*-difluorobenzene (DFB) for subsequent kinetic resolution polymerization.

## 7. Outlook

Over the past few several years, Lewis pair polymerization catalysis has attracted an explosive level of interest in the field of polymer chemistry. Lewis pairs have higher thermal stability to enable ROP under industrially relevant melt processing conditions. Exciting opportunities still exist for the ROP of cyclic ester using Lewis pairs, for example, the degree of polymerization control needs significant improvement, the molecular weight of the polyester is still limited, addressing challenges of designing Lewis pair catalysts that can mediate stereocontrolled ROP of lactide and related cyclic esters at ambient temperature and above, and detailed mechanism need be studied further in detail.

## References

[1] Dechy-Cabaret O., Martin-Vaca B., Bourissou D. (2004). Controlled ring-opening polymerization of lactide and glycolide. Chem. Rev..

[2] Wu J.C., Yu T.L., Chen C.T., Lin C.C. (2006). Recent developments in main group metal complexes catalyzed/initiated polymerization of lactides and related cyclic esters. Coord. Chem. Rev..

[3] O’Keefe B.J., Hillmyer M.A., Tolman W.B. (2001). Polymerization of lactide and related cyclic esters by discrete metal complexes. J. Chem. Soc. Dalton Trans..

[4] Dove A.P. (2012). Organic catalysis for ring-opening polymerization. ACS Macro Lett..

[5] Kamber N.E., Jeong W., Waymouth R.M., Pratt R.C., Lohmeijer B.G.G., Hedrick J.L. (2007). Organocatalytic ring-opening polymerization. Chem. Rev..

[6] Ottou W.N., Sardon H., Mecerreyes D., Vignolle J., Taton D. (2016). Update and challenges in organo-mediated polymerization reactions. Prog. Polym. Sci..

[7] Piedra-Arroni E., Brignou P., Amgoune A., Guillaume S.M., Carpentier J.F., Bourissou D. (2011). A dual organic/organometallic approach for catalytic ring-opening polymerization. Chem. Commun..

[8] Kreitner C., Geier S.J., Stanlake L.J., Caputo C.B., Stephan D.W. (2011). Ring openings of lactone and ring contractions of lactide by frustrated lewis pairs. Dalton Trans..

[9] Piedra-Arroni E., Ladaviere C., Amgoune A., Bourissou D. (2013). Ring-opening polymerization with Zn(C_6_F_5_)_2_-based lewis pairs: Original and efficient approach to cyclic polyesters. J. Am. Chem. Soc..

[10] Li X.Q., Wang B., Ji H.Y., Li Y.S. (2016). Insights into the mechanism for ring-opening polymerization of lactide catalyzed by Zn(C_6_F_5_)_2_/organic superbase lewis pairs. Catal. Sci. Technol..

[11] Schnee G., Fliedel C., Avilés T., Dagorne S. (2013). Neutral and cationic *N*-heterocyclic carbene zinc adducts and the BnOH/Zn(C_6_F_5_)_2_ binary mixture-characterization and use in the ring-opening polymerization of β-butyrolactone, lactide, and trimethylene carbonate. Eur. J. Inorg. Chem..

[12] Zhang Y., Miyake G.M., John M.G., Falivene L., Caporaso L., Cavallo L., Chen E.Y.X. (2012). Lewis pair polymerization by classical and frustrated lewis pairs: Acid, base and monomer scope and polymerization mechanism. Dalton Trans..

[13] Gruseck U., Heuschmann M. (1987). 2-Alkylidenimidazolidine-Synthese, Basizitat, ^1^H- und ^13^C-NMR-Spektren. Eur. J. Inorg. Chem..

[14] Kronig S., Jones P.G., Tamm M. (2013). Preparation of 2-alkylidene-substituted 1,3,4,5-tetramethylimidazolines and their reactivity towards Rh^I^ complexes and B(C_6_F_5_)_3_. Eur. J. Inorg. Chem..

[15] Powers K., Hering-Junghans C., McDonald R., Ferguson M.J., Rivard E. (2016). Improved synthesis of *N*-heterocyclic olefins and evaluation of their donor strengths. Polyhedron.

[16] Imbrich D.A., Frey W., Naumann S., Buchmeiser M.R. (2016). Application of imidazolinium salts and *N*-heterocyclic olefins for the synthesis of anionic and neutral tungsten imido alkylidene complexes. Chem. Commun..

[17] Al-Rafia S.M., Malcolm A.C., Liew S.K., Ferguson M.J., McDonald R., Rivard E. (2011). Intercepting low oxidation state main group hydrides with a nucleophilic *N*-heterocyclic olefin. Chem. Commun..

[18] Blumel M., Noy J.M., Enders D., Stenzel M.H., Nguyen T.V. (2016). Development and applications of transesterification reactions catalyzed by *N*-heterocyclic olefins. Org. Lett..

[19] Wang Y.B., Wang Y.M., Zhang W.Z., Lu X.B. (2013). Fast CO_2_ sequestration, activation, and catalytic transformation using *N*-heterocyclic olefins. J. Am. Chem. Soc..

[20] Jia Y.B., Wang Y.B., Ren W.M., Xu T., Wang J., Lu X.B. (2014). Mechanistic aspects of initiation and deactivation in *N*-heterocyclic olefin mediated polymerization of acrylates with alane as activator. Macromolecules.

[21] Naumann S., Thomas A.W., Dove A.P. (2015). *N*-heterocyclic olefins as organocatalysts for polymerization: Preparation of well-defined poly(propylene oxide). Angew. Chem. Int. Ed..

[22] Saptal V.B., Bhanage B.M. (2016). *N*-heterocyclic olefins as robust organocatalyst for the chemical conversion of carbon dioxide to value-added chemicals. ChemSusChem..

[23] Blümel M., Crocker R.D., Harper J.B., Enders D., Nguyen T.V. (2016). *N*-heterocyclic olefins as efficient phase-transfer catalysts for base-promoted alkylation reactions. Chem. Commun..

[24] Wang Q., Zhao W., He J., Zhang Y., Chen E.Y.X. (2017). Living ring-opening polymerization of lactones by *N*-heterocyclic olefin/Al(C_6_F_5_)_3_ lewis pairs: Structures of intermediates, kinetics, and mechanism. Macromolecules.

[25] Nakayama Y., Kosaka S., Yamaguchi K., Yamazaki G., Tanaka R., Shiono T. (2017). Controlled ring-opening polymerization of l-lactide and ε-caprolactone catalyzed by aluminum-based lewis pairs or lewis acid alone. J. Polym. Sci. Part A Polym. Chem..

[26] Schnee G., Bolley A., Hild F., Specklin D., Dagorne S. (2017). Group 13 metal (Al, Ga, In) alkyls supported by *N*-heterocycliccarbenes for use in lactide ring-opening polymerization catalysis. Catal. Today.

[27] Naumann S., Scholten P.B., Wilson J.A., Dove A.P. (2015). Dual catalysis for selective ring-opening polymerization of lactones: Evolution toward simplicity. J. Am. Chem. Soc..

[28] Wilson J.A., Hopkins S.A., Wright P.M., Dove A.P. (2014). ‘Immortal’ ring-opening polymerization of ω-pentadecalactone by Mg(BHT)_2_(THF)_2_. Polym. Chem..

[29] Naumann S., Wang D. (2016). Dual catalysis based on *N*-heterocyclic olefins for the copolymerization of lactones: High performance and tunable selectivity. Macromolecules.

[30] Walther P., Naumann S. (2017). *N*-heterocyclic olefin-based (co)polymerization of a challenging monomer: Homopolymerization of ω-pentadecalactone and its copolymers with γ-butyrolactone, δ-valerol-actone, and ε-caprolactone. Macromolecules.

[31] Hong M., Chen E.Y. (2016). Completely recyclable biopolymers with linear and cyclic topologies via ring-opening polymerization of gamma-butyrolactone. Nat. Chem..

[32] Zhu J.B., Chen E.Y. (2015). From meso-lactide to isotactic polylactide: Epimerization by B/N lewis pairs and kinetic resolution by organic catalysts. J. Am. Chem. Soc..

